# Deuterium-oxide-assisted stimulated Raman scattering microscopy: metabolic imaging for precision medicine

**DOI:** 10.1117/1.BIOS.2.4.040901

**Published:** 2025-10-03

**Authors:** Yuhui Li, Shuhua Yue

**Affiliations:** Key Laboratory of Biomechanics and Mechanobiology (Beihang University), Ministry of Education; Key Laboratory of Innovation and Transformation of Advanced Medical Devices, Ministry of Industry and Information Technology; National Medical Innovation Platform for Industry-Education Integration in Advanced Medical Devices (Interdiscipline of Medicine and Engineering); Beihang University, School of Biological Science and Medical Engineering, Beijing, China

**Keywords:** Stimulated Raman scattering imaging, deuterium oxide, precision medicine, metabolism

## Abstract

**Significance:**

Metabolomics is transforming personalized medicine by enabling the customization of treatment plans based on the unique metabolic profiles of individual patients. However, general metabolomics technologies are destructive and lack spatial resolution, which limits their application in live-cell analysis and dynamic imaging studies. Furthermore, these methods typically lack sufficient spatial resolution, which limits their capacity to capture metabolic heterogeneity at the single-cell or subcellular level. These limitations hinder the study of spatially and temporally dynamic metabolic processes in diseases such as cancer, metabolic, and neurological disorders.

**Aim:**

Our review summarizes advances in deuterium-oxide-assisted stimulated Raman scattering ($D_{2}O\text{-}\mathrm{SRS}$) microscopy, with a focus on its capabilities for real-time, high-resolution metabolic imaging. We discuss how this technology enables real-time tracking of biosynthetic processes in living systems, contributing to the advancement of precision diagnostics and therapeutic strategies, highlighting its recent progress in tumor metabolism monitoring, and drug sensitivity testing for infectious diseases, as well as its future potential applications.

**Approach:**

We outline the nonlinear optical principles of SRS, the deuterium oxide ($D_{2}O$) labeling strategy that generates carbon–deuterium (C–D) vibrational signals in newly synthesized macromolecules, and key applications in tracking lipid, protein, and nucleic acid biosynthesis.

**Results:**

$D_{2}O\text{-}\mathrm{SRS}$ enables real-time, quantitative imaging of metabolic activity with subcellular resolution and minimal perturbation, offering chemical and spatial insight into metabolic dynamics.

**Conclusions:**

$D_{2}O\text{-}\mathrm{SRS}$ microscopy represents a significant advancement in live metabolic imaging, offering a powerful tool for investigating disease-related metabolic dysfunction. This technology enables high-resolution, real-time metabolic imaging in living systems. As a result, $D_{2}O\text{-}\mathrm{SRS}$ is expected to promote the transformation and implementation of basic metabolic research into clinical applications.

Statement of Discovery $D_{2}O\text{-}\mathrm{SRS}$ enables real-time quantitative imaging of metabolic activity at subcellular resolution with minimal perturbation, thereby allowing visualization of the metabolic dynamics of biomolecules, including lipids, proteins, and nucleic acids. $D_{2}O\text{-}\mathrm{SRS}$ is expected to evolve into a “metabolic microscope” that enables dynamic analysis of physiological processes and supports accurate disease diagnosis, thereby facilitating the translation of fundamental metabolic research into clinical practice.

## 1 Introduction

### 1.1 Metabolic Profiling in Precision Medicine

Precision medicine refers to treatment strategies that are specifically tailored to individual patients based on a molecular understanding of their disease phenotype.^1^^–^^3^ The foundation of precision medicine lies in the identification of key biomarkers through various technologies, including genomics, proteomics, and metabolomics. These technologies provide insights into disease mechanisms and help identify potential therapeutic targets, which can then be validated and applied to large sample populations.^4^^,^^5^ Among these approaches, the synergistic application of multi-omics is pivotal for uncovering disease-related metabolic changes.^6^ Specifically, metabolomics stands out as a cornerstone for diagnosis, prognosis, and treatment monitoring, as disruption of metabolic pathways results in specific metabolite imbalances that are widely acknowledged as key indicators of disease.^7^ These profiles closely reflect the phenotypic information of the subject and facilitate disease diagnosis, prognostic evaluation, and monitoring of therapeutic responses.^8^

Cellular metabolism is a highly dynamic and heterogeneous process that varies across spatial and temporal scales. In various diseases, this heterogeneity is particularly pronounced: within tumors, remodeling of the microenvironment by hypoxia and nutrient deprivation produces spatially divergent metabolic states that critically shape tumor evolution, whereas in infectious diseases, pathogen-driven remodeling of the microenvironment creates spatially variable metabolic states that strongly influence disease progression.^9^^–^^12^ Although metabolomics techniques can classify thousands of metabolites, these approaches typically rely on population-averaged measurements, which lack the spatial resolution required to resolve metabolic activities at the subcellular level.^13^^,^^14^ Single-cell sequencing–based technologies are capable of providing valuable insights into cellular heterogeneity, but they remain insufficient for capturing the spatial heterogeneity present in complex disease tissues.^15^^,^^16^ Taken together, these limitations underscore the urgent need for advanced metabolic imaging techniques that integrate high specificity, as well as temporal and spatial resolution, to achieve a more comprehensive understanding of disease development and progression.

In terms of current spatial omics techniques, such as spatial transcriptomics and mass spectrometry imaging, these offer promising solutions by linking molecular data with tissue architecture. However, these methods often involve complex sample preparation processes, are prone to potential sample damage, and are typically expensive. Moreover, their resolution is insufficient to study subcellular structures in detail. This limitation necessitates the advancement of spatial omics technologies that are capable of generating high-resolution metabolic data at single-cell and subcellular resolution across heterogeneous cellular regions. Raman scattering microscopy, as a label-free, high-resolution imaging technique, holds great promise in this regard. It provides a non-perturbative approach to studying metabolic activity at the single-cell and subcellular levels, enabling real-time monitoring of disease progression and therapeutic responses with high spatial resolution.

### 1.2 Principle of the Raman Scattering Process

**Fig. 1:**
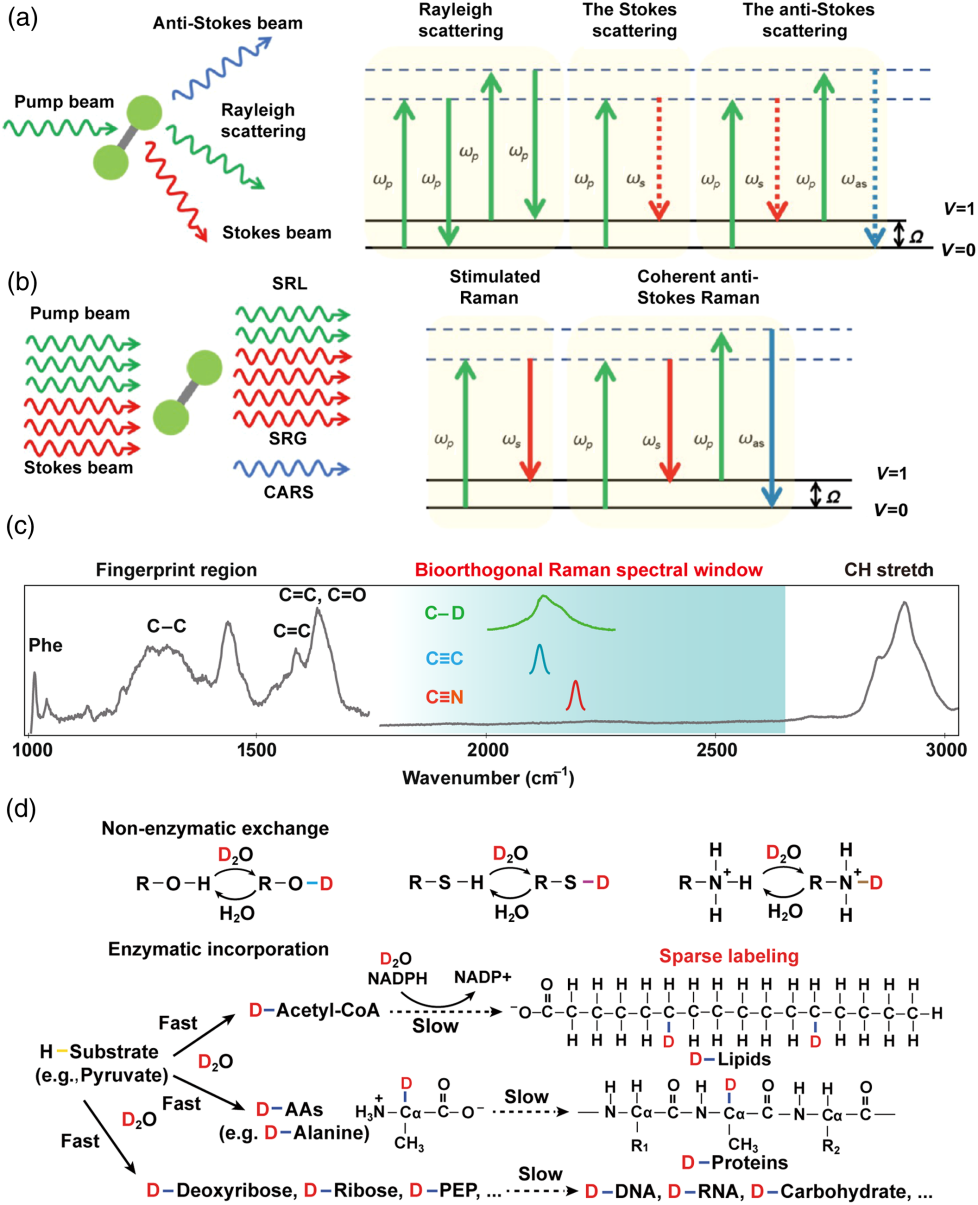
Principle of the Raman scattering process and Raman probes. (a) Principle of the spontaneous Raman scattering process. (b) Principle of coherent Raman scattering process. Reprinted with permission from Ref. 7. (c) Typical cell Raman spectrum with the cell spectral-silent window where no Raman peak from endogenous biomolecules appears. (d) $D_{2}O$-derived deuterium can form O–D, S–D, and N–D bonds through reversible non-enzymatic H/D exchange and be incorporated into C–D bonds of metabolic precursors for the synthesis of macromolecules through irreversible enzymatic incorporation. Note that, under the condition of sparse labeling, the position of deuterium labeling on lipids is random, and this illustration only represents one possible labeling pattern. Reprinted with permission from Ref. 19.

Owing to high chemical selectivity, subcellular spatial resolution, and non-invasiveness, spontaneous Raman scattering microscopy is widely used for metabolite detection in single cells.^17^^,^^18^ Unfortunately, the spontaneous Raman scattering process, illustrated in Fig. 1(a), has an extremely small cross-section ($10^{-30}\;{\mathrm{cm}}^{2}$) per molecule.^7^ Consequently, it takes approximately tens of minutes per frame, which hinders its application in capturing dynamic processes in living systems. To enhance the Raman scattering signal, coherent Raman scattering (CRS) microscopy was developed; the principle of the CRS process is depicted in Fig. 1(b).^7^ Specifically, as nonlinear optical processes of coherent excitation, the large signal level in CRS microscopy enables high-speed imaging, vibrational activation rates of CRS can be drastically accelerated by $10^{8}$ compared with that of the inherently weak spontaneous Raman scattering. Representative techniques of CRS include coherent anti-Stokes Raman scattering (CARS) and SRS. CARS relies on a four-wave mixing process that generates signals at a new frequency distinct from the excitation beams. However, its performance is constrained by a strong non-resonant background, which distorts spectral features and complicates quantitative analysis. In particular, for weaker Raman bands, the CARS signal is often buried in a large non-resonant background contributed by the medium.^20^^,^^21^

To further enhance sensitivity and specificity, SRS microscopy was developed in 2008 and for the first time applied to label-free biomedical imaging.^22^ In contrast to CARS, the SRS signal is intrinsically free of non-resonant background and provides spectral profiles that are nearly identical to the spontaneous Raman spectrum.^23^ Moreover, the SRS intensity is linearly dependent on molecular concentration, which enables straightforward interpretations of chemical mapping to generate quantitative concentration maps of targeted biomolecules.^24^ By exploiting the intrinsic Raman spectra of biomolecules, label-free SRS microscopy with chemical bond specificity is routinely utilized to map a range of metabolites and drugs, reveal their spatial distribution in living cells and tissues, and enable biomarker-based diagnostics.^25^^,^^26^ Label-free SRS imaging of chemical bond vibrations primarily focuses on the C–H stretching region (2800 to $3100\;{\mathrm{cm}}^{-1}$), because of the strong signals from lipids and proteins.^27^ However, these chemical bonds are commonly shared by different endogenous molecules, which constrains the molecular specificity and sensitivity of label-free SRS imaging.^28^

### 1.3 Deuterium Probes and the Promise of D_2_O-SRS Imaging

Raman probes have been developed to exploit the advantages of the cellular silent Raman window (1800 to $2600\;{\mathrm{cm}}^{-1}$), which is free of endogenous molecular vibrations. Figure 1(c) illustrates a typical cell Raman spectrum that highlights this spectral-silent region.^29^ By introducing specific chemical bonds, these probes effectively minimize background interference and enable highly sensitive and specific detection of target molecules. Moreover, Raman probes not only help to elucidate the spatial distribution of biomolecules but also provide insights into the metabolic turnover of labeled species within cells, a critical capability absent in label-free imaging.^30^^,^^31^ Over the past decades, significant advancements have been made in the development of these probes.^32^^–^^34^ Specifically, probes for SRS imaging are categorized into three types based on size and application: vibrational probes for small molecule imaging (<1  nm), Raman dye palettes for super-multiplex imaging (<10  nm), and Raman-active nanomaterials (>10  nm).^35^^,^^36^

Among the various vibrational probes, $D_{2}O$ has gained significant attention due to its cost-effectiveness and excellent compatibility with biological systems. $D_{2}O$ labeling involves replacing hydrogen with deuterium in the C–H bonds of biomolecules, such as nucleotides, proteins, lipids, and carbohydrates, as illustrated in Fig. 1(d).^19^ This subtle isotopic substitution, when coupled with SRS microscopy, enables dynamic observation of metabolic processes without disrupting cellular functions.^29^ These techniques provide valuable temporal insights into processes such as amino acid and lipid turnover, which cannot be captured using label-free imaging methods.^37^ As a result, $D_{2}O$ labeling has provided critical information for understanding cellular metabolism. The technological advancements in SRS microscopy and the development of vibrational probes for biological applications have been thoroughly explored in previous reviews.^38^^,^^39^

## 2 Advancements in D_2_O-SRS: Focus on Biomolecular Imaging

### 2.1 Principle of D_2_O Probe

Water is an essential component of organisms, participating in metabolic pathways such as the synthesis of nucleic acids, lipids, and proteins. For imaging these processes, $D_{2}O$, a stable isotopic form of water, serves as a critical tracer. $D_{2}O$ retains the biochemical functionality of water while introducing detectable deuterium labels into biomolecules, enabling real-time tracking of metabolic activity.^40^ In biological systems, $D_{2}O$ rapidly equilibrates with the total water levels, forming a variety of deuterium (D)-containing biomolecules through non-enzymatic H/D exchange and enzymatic binding. These labeled precursors are metabolized into macromolecules such as nucleic acids and lipids.^41^^–^^43^

### 2.2 Raman Spectroscopy for Distinguishing Deuterium Incorporation

Raman spectroscopy provides a noninvasive, optical approach to distinguish metabolic incorporation from non-enzymatic exchange *in situ*, because various X–D bonds have intrinsically distinct stretching vibrational features. Raman spectra of carbon–deuterium (C–D) bonds are clearly separated from those of C–H, the O–D in $D_{2}O$ as well as non-enzymatically formed O–D, S–D, and N–D bonds. This allows the direct detection of biosynthetic incorporation of deuterium through the amount of C–D bonds. Importantly, the C–D bond also exhibits vibrational signatures that are spectrally distinct from endogenous vibrational modes of cellular biomolecules, which further reduces spectral interference and improves detection specificity and sensitivity. In fact, spontaneous Raman spectroscopy has recently been employed to identify metabolic activity in bacteria after $D_{2}O$ treatment.^44^^,^^45^ However, the technique’s low sensitivity—due to weak Raman scattering—often necessitates large sample volumes or prolonged integration times. Moreover, background interference from substrates complicates bacterial signal detection. This approach also has difficulties in generating spatially resolved images due to slow imaging speed. These limitations have driven the adoption of advanced techniques such as SRS.^46^

### 2.3 Advances of D_2_O-SRS Imaging

Compared with spontaneous Raman spectroscopy, SRS microscopy is an emerging nonlinear Raman imaging technology with a substantial sensitivity boost through quantum amplification by stimulated emission, which enables orders of magnitude faster acquisition time, fine spectral resolution, compatibility with fluorescence, and three-dimensional (3D) optical sectioning capability in tissues and even living animals.^22^^,^^28^^,^^47^ These unique advantages of SRS microscopy, combined with the chemical features of the C–D vibrational spectrum, led to the development of $D_{2}O\text{-}\mathrm{SRS}$ microscopy, which uses $D_{2}O$ as an imaging contrast agent to specifically trace lipid, protein, and DNA metabolism in cells and tissues.

Compared with other Raman probes, $D_{2}O$ offers several practical advantages, particularly its ability to quantify metabolic turnover rates *in situ* with minimal cytotoxicity and broad compatibility across organisms and tissue types. In addition, $D_{2}O$ is cost-effective and easy to administer, typically through simple incubation or systemic delivery, making it well-suited for both *in vitro* and *in vivo* studies.^29^ In comparison, alkyne-based probes provide higher chemical specificity and signal intensity when targeting a particular biosynthetic process, but their applicability is often restricted to specific pathways and may require sophisticated probe design. Similarly, isotope-labeled amino acids or glucose probes can be used for targeted studies, but they usually involve complex synthesis or may affect metabolic balance when applied in high concentrations.

In terms of application scenarios, $D_{2}O$-SRS imaging has shown significant promise in cancer biology and neurodegenerative diseases, particularly in tracking metabolic reprogramming in tumors. Shi et al.^19^ first proposed the $D_{2}O\text{-}\mathrm{SRS}$ technology, which enabled high-resolution visualization of lipid, protein, and DNA metabolism in living organisms. This provided an important tool for revealing metabolic reprogramming of tumors, the activity of infectious pathogens, and other metabolic abnormalities related to diseases. Moreover, $D_{2}O$ labeling enables real-time mapping of lipid synthesis dynamics at the subcellular level, which has been applied to identify aggressive tumor phenotypes and monitor treatment responses.^19^ Furthermore, in infectious disease models, $D_{2}O\text{-}\mathrm{SRS}$ imaging has been used to assess the metabolic activity of pathogens. Taken together, the distinctive features of $D_{2}O\text{-}\mathrm{SRS}$ have become a powerful and versatile tool for metabolic research.

## 3 Detection of Biomolecular Metabolic Dynamics in Infectious Disease

### 3.1 Bacterial Infectious Diseases

Bacterial infections are a leading cause of human diseases, and antibiotics have been developed to target bacterial pathogens. However, the misuse and overuse of antibiotics have led to the rise of antimicrobial resistance, which has become a significant public health threat, causing ∼1 million deaths globally each year.^48^ Moreover, untreated bacterial infections can lead to conditions such as pneumonia, urinary tract infections, peritonitis, sepsis, and more. Patients in the ICU, in particular, often have weakened immune systems, making them more susceptible to infections such as *Klebsiella pneumoniae*, which can be life-threatening if not treated promptly.^49^ Therefore, to combat antibiotic resistance, it is crucial to obtain rapid information on bacterial susceptibility to antibiotics. Rapid antimicrobial susceptibility testing (AST) not only enables more effective patient treatment but also minimizes the overuse of antibiotics, potentially reducing long-term side effects.^50^^,^^51^

**Fig. 2:**
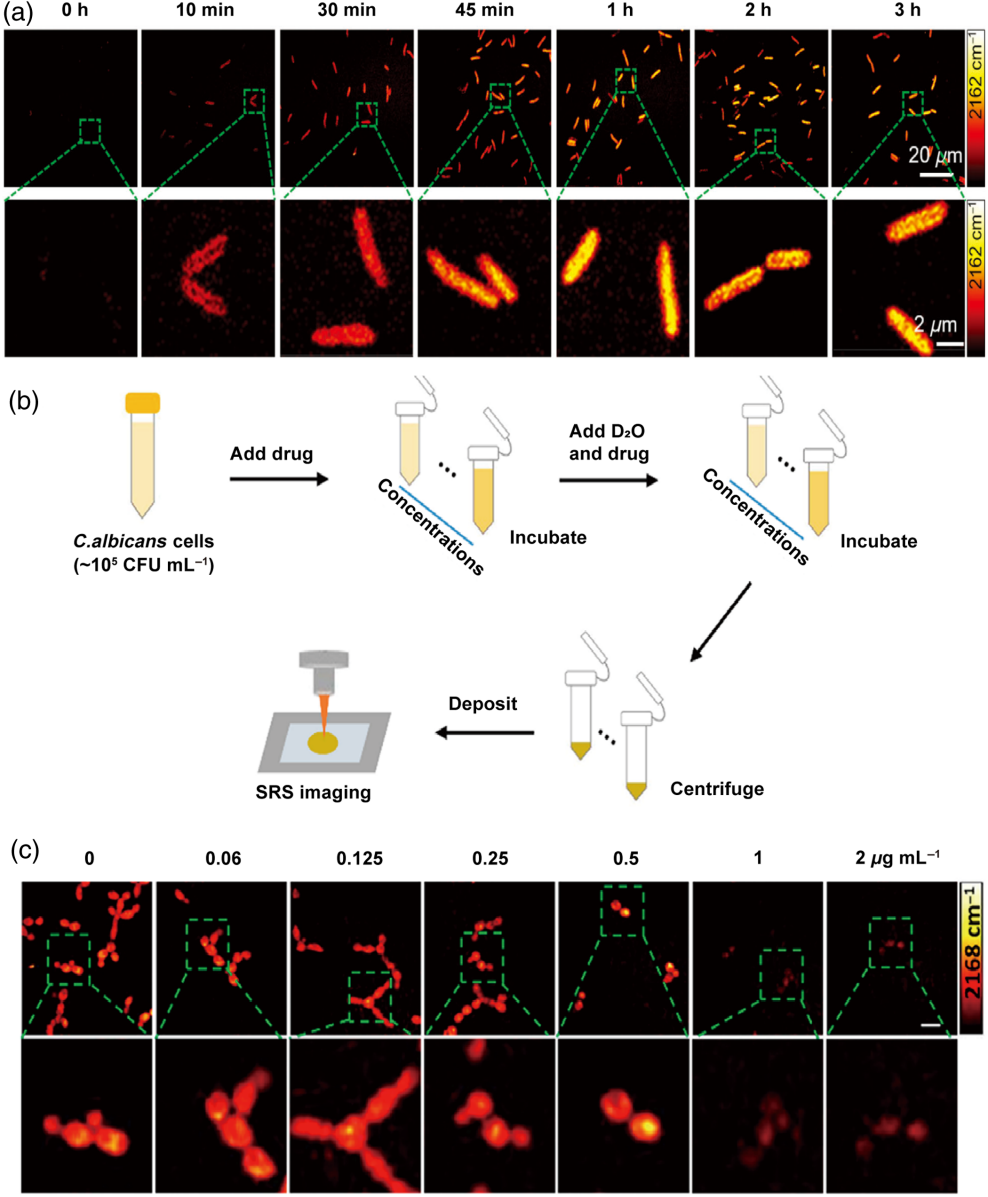
$D_{2}O\text{-}\mathrm{SRS}$ imaging of biomolecular metabolic dynamics in infectious diseases. (a) Time-lapse SRS imaging of *P. aeruginosa* after culture in $D_{2}O$-containing medium. Reprinted with permission from Ref. 38. (b) Establishing a model for rapid AFST to amphotericin B, and workflow of rapid AFST by $D_{2}O\text{-}\mathrm{SRS}$ metabolic imaging. (c) SRS images of a susceptible *C. albicans* strain treated with different concentrations of amphotericin B at the C–D frequency. Scale bar: 20  μm. Reprinted with permission from Ref. 52.

Conventional AST methods, which often require 24 to 48 h for results, are ill-suited to address urgent clinical decisions. $D_{2}O\text{-}\mathrm{SRS}$ technology overcomes this limitation by enabling real-time monitoring of bacterial metabolic changes, providing rapid insights into antibiotic susceptibility. Zhang et al. first produced AST results by femtosecond SRS imaging of $D_{2}O$ metabolism. Metabolic incorporation of $D_{2}O$ into biomass in a single bacterium and the metabolic response to antibiotics are probed in as short as 10 min after culture in 70% $D_{2}O$ medium, as illustrated in Fig. 2(a), the fastest among current technologies.^38^ This approach allows for obtaining single-cell metabolism inactivation concentration (SC-MIC) within less than 2.5 h from colony to results, aiding clinicians in rapidly diagnosing bacterial infections and selecting effective antibiotics.^38^ Moreover, Liang et al.^49^ evaluated $D_{2}O$–labeled single-cell Raman spectroscopy on 29 *Klebsiella pneumoniae* strains isolated from ICU patients. They reported that the susceptibility results from this method were consistent with routine AST outcomes in approximately 90% of cases. Although the method was not directly used to guide clinical treatment in this study, these findings suggest that, with further improvements, $D_{2}O\text{-}\mathrm{SRS}$ has the potential to provide rapid preliminary AST results and may offer clinical benefit in the future.^49^ Furthermore, Zhang et al.^53^ quantified bacterial metabolic activity by measuring the C–D bond concentration in bacteria after $D_{2}O$ incubation. They also analyzed bacterial morphological deformations caused by β-lactam antibiotics.^53^ Using these two markers, they developed an effective method for performing AST of cefotaxime on 103 *E. coli* strains, achieving a 93.2% categorical agreement with standard reference methods. This combination of metabolic activity and morphological information provides a rapid and reliable way to conduct AST, underscoring its reliability for clinical adoption.

Despite the advances of $D_{2}O\text{-}\mathrm{SRS}$ in accelerating AST, challenges remain in achieving both rapid and accurate bacterial identification. To address this, Zhang et al.^54^ developed a composite Raman microscopy (CRM) system that combines Raman spectroscopy and SRS microscopy. CRM utilizes the chemical selectivity of Raman spectroscopy and the high-throughput nature of SRS imaging to identify bacterial pathogens and perform AST. The authors trained a deep learning model on an extensive bacterial Raman spectra dataset to identify six common bacterial pathogens and used $D_{2}O\text{-}\mathrm{SRS}$ to quantify metabolic activity for AST. The system performed Gram-staining classification directly from urine samples in less than 0.5 h and completed AST in under 2.5 h.^54^ By shortening the diagnostic time for urinary tract infections, this approach has the potential to optimize antibiotic use, which may in turn improve treatment outcomes and reduce healthcare costs. Taken together, these studies suggest that $D_{2}O\text{-}\mathrm{SRS}$ is able to accelerate AST and improve precision by integrating metabolic and structural biomarkers, providing a potential tool for combating antibiotic resistance in clinical care settings.

Gut microbiota play a crucial role in maintaining human health. They not only participate in basic physiological functions such as digestion, immunity, and metabolism but also indirectly regulate the overall health by influencing multiple aspects including the nervous system. Studies have found that the imbalances of gut microbiota are closely related to the occurrence of various diseases such as obesity, diabetes, depression, and other diseases.^55^^,^^56^ To explore gut microbiota dynamics, Hong et al.^46^ developed a rapid AST method by using hyperspectral stimulated Raman scattering (hSRS) imaging to monitor the glucose metabolic activity of live bacterial single cells. Using vancomycin-susceptible and -resistant *E. faecalis* as models, they demonstrated that hyperspectral SRS imaging can quantitatively monitor the metabolic uptake of deuterated glucose by individual live bacteria. Notably, the metabolic activity response of the sensitive strain to antibiotics differed from that of the resistant strain within only 0.5 h after the addition of antibiotics. Therefore, this method can determine the bacterial sensitivity and the minimum inhibitory concentration (MIC) of antibiotics within one cell cycle. This metabolic imaging method is applicable to different bacterial species, including *E. coli*, *K. pneumoniae*, and *S. aureus* as well as different antibiotics.^46^

One of the primary challenges in microbiome research, particularly in environmental and medical samples, is understanding the functional properties of microbial community members at the single-cell level. Traditional experimental methods for studying the functions of individual microorganisms in their native habitats are time-consuming and typically allow for the analysis of only a few cells or samples. Single-cell isotope probing has emerged as a key tool to address this challenge; however, current detection methods for determining isotope incorporation into single cells lack high-throughput capabilities.^57^^,^^58^ To deal with the challenges in detecting low concentrations of metabolites inside small cells with diameters around 1  μm, Ge et al.^59^ developed a high-throughput imaging-based approach termed stimulated Raman scattering–two-photon fluorescence *in situ* hybridization (SRS-FISH). This platform enables metabolic and identity analyses of microbial communities with single-cell resolution. This platform maximizes the isotope label content in cells and exploits the intense SRS signal from the Raman band used for isotope detection. Conventionally, FISH is performed separately by one-photon excited fluorescence microscopy.^59^ To enhance efficiency, they developed a system that implements highly sensitive SRS metabolic imaging with two-photon FISH using the same laser source. These efforts collectively led to a high-throughput platform that enables correlative imaging of cell identity and metabolism at a speed of 10 to 100 ms per cell. In comparison, it takes about 20 s to record a Raman spectrum from a single cell in a conventional spontaneous Raman FISH experiment. Using this technique, the authors delineated the metabolic responses of over 30,000 individual cells to various mucosal sugars in the human gut microbiome by incorporating deuterium from heavy water as an activity marker.^59^ This study demonstrates the sensitivity and speed of SRS-FISH, offering new insights into the fine temporal, spatial, and individual activity patterns of microbial cells within complex communities.

In recent years, $D_{2}O\text{-}\mathrm{SRS}$ technology has shown significant potential in detecting bacterial antimicrobial resistance, diagnosing fungal infections, and studying intestinal microbiota. Although these studies have provided proof of principles, there are some limitations that need to be addressed for clinical translations, including the labor-intensive imaging requirements. The current SRS-FISH technique necessitates considerable manual intervention. To improve throughput and automation, future developments should focus on integrating more efficient sample production processes and optimizing data acquisition protocols to enable the automatic imaging and analysis of multiple samples. In addition, current research is predominantly focused on model strains and small-scale samples, with limited direct testing on patient samples and validation across a wider sample range. To accelerate clinical translation, future studies should prioritize the application of this technology to patient samples, establish a standardized sample production process to enhance the speed of sample testing, and implement robust quality control systems to ensure the reliability and reproducibility of results.

### 3.2 Fungal Infectious Diseases

Fungal infections cause more than 1.5 million deaths worldwide each year. Of particular concern is invasive candidiasis, a bloodstream infection, which is a growing concern in health care settings due to its high morbidity and mortality rates.^52^ Although antifungal drug susceptibility testing (AFST) plays a key role in predicting pathogen response to antifungal treatment, the gold standard AFST based on the broth microdilution (BMD) method takes at least 24 h due to the slow growth rate of fungal cells. This delay significantly hampers timely treatment decisions, making it difficult to control the spread of infections. Commercial methods such as Etest and VITEK 2 Yeast System, although more efficient than BMD, still require 12 to 48 h to yield results. The urgent need for faster, more efficient AFST methods is exacerbated by the growing threat of antifungal resistance.^60^^,^^61^ Although genotypic methods such as nucleic acid diagnostics and matrix-assisted laser desorption time-of-flight mass spectrometry (MALDI-TOF MS) can reduce testing time, they typically cover only a limited spectrum of microorganisms and do not effectively determine the minimum inhibitory concentration (MIC).^62^^,^^63^

In this context, $D_{2}O\text{-}\mathrm{SRS}$ offers a promising solution by providing both rapid results and functional insights into microbial viability. For instance, Chen et al.^52^ utilized $D_{2}O\text{-}\mathrm{SRS}$ to measure metabolic activity in individual fungal cells, as illustrated in Figs. 2(b) and 2(c), marking a significant advancement in rapid AFST. This approach showed that metabolic changes in *Candida albicans* in response to antifungal drugs could be detected within just 4 h, with the results fully consistent with those obtained using the gold standard BMD method. This reduction in testing time could significantly enhance clinical decision-making, enabling faster diagnoses and treatment adjustments.^52^

## 4 Detection of Biomolecular Metabolic Dynamics in Cancer

Metabolic reprogramming in cancer cells drives their growth, proliferation, and migration, among other critical biological processes. Understanding these metabolic alterations is key to uncovering the mechanisms underlying cancer development and identifying potential therapeutic targets.^7^^,^^64^ $D_{2}O\text{-}\mathrm{SRS}$ overcomes many of the limitations of traditional metabolic analysis methods. By exploiting the properties of heavy water, SRS enables real-time imaging with high sensitivity and spatial resolution, capturing transient changes in metabolic dynamics within cancer cells. Beyond mapping spatial distributions, $D_{2}O\text{-}\mathrm{SRS}$ quantifies reaction rates and pathway fluxes, revealing how metabolic rewiring in cancer cells sustains proliferation or evades therapies, enabling researchers to more comprehensively understand the metabolic mechanisms of cancer cells.^65^

### 4.1 Breast Cancer

**Fig. 3:**
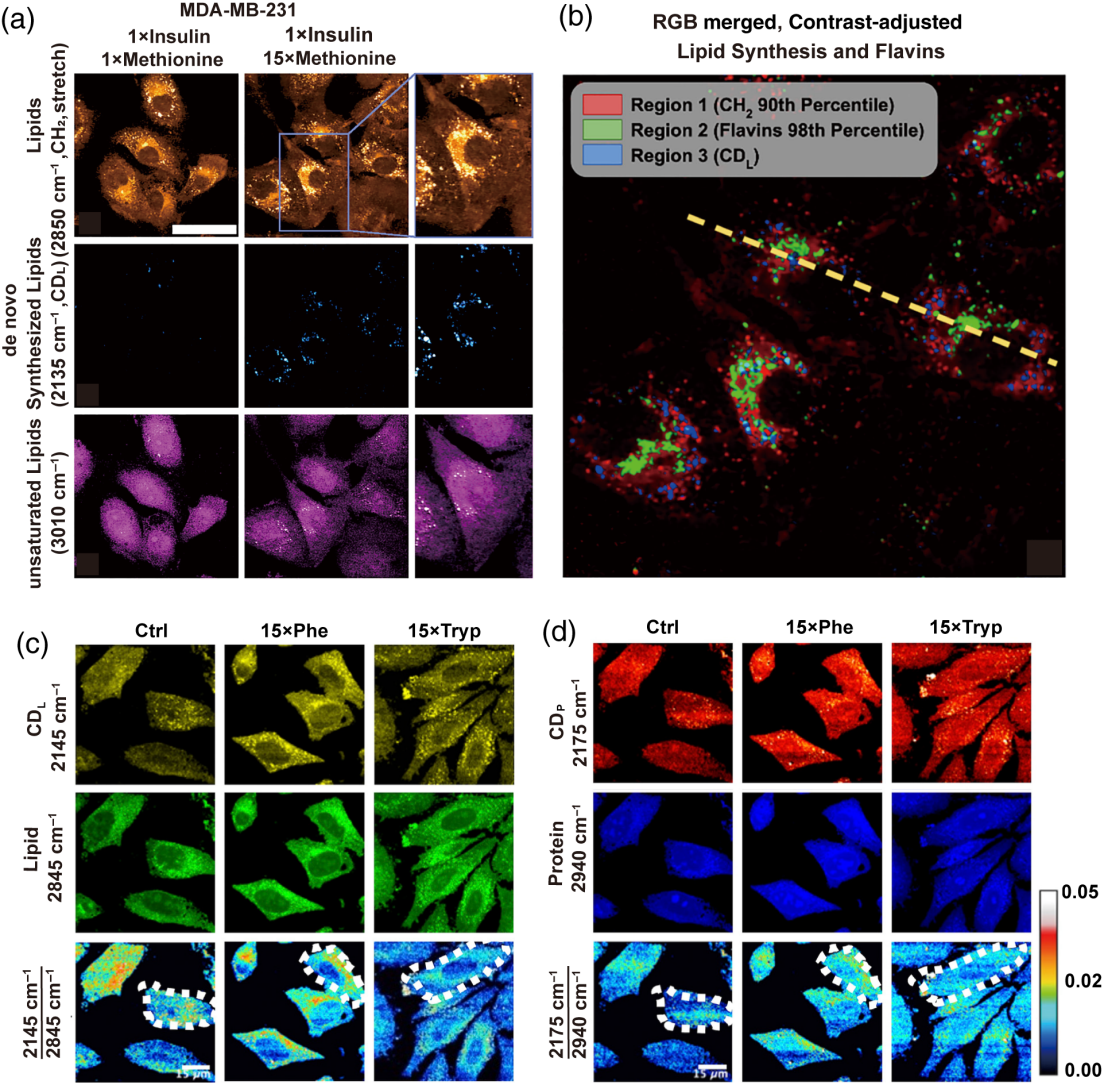
Multi-modal optical analysis depicts tumor metabolic phenotypes. (a) Multi-channel images illustrate SRS and TPEF image channels of interest for lipidomic responses to excess methionine, lipid (${\mathrm{CH}}_{2}$; $2845\;{\mathrm{cm}}^{-1}$), $D_{2}O$-labeled lipid (${\mathrm{CD}}_{L}$; $2135\;{\mathrm{cm}}^{-1}$). (b) Overlaid composite regions image of the 15× methionine lipid (${\mathrm{CH}}_{2}$), flavins, and *de novo* synthesized lipids (${\mathrm{CD}}_{L}$). Channels were masked according to the indicated thresholds using ImageJ, and contrast was adjusted for optimal clarity. Reprinted with permission from Ref. 67. (c), (d) $D_{2}O\text{-}\mathrm{SRS}$ microscopy visualizes, deuterium-labeled lipid (${\mathrm{CD}}_{L}$; ${2145\;\mathrm{cm}}^{-1}$), deuterium-labeled protein (${\mathrm{CD}}_{P}$; $2175\;{\mathrm{cm}}^{-1}$), and protein (${\mathrm{CH}}_{3}$; $2940\;{\mathrm{cm}}^{-1}$) channels in HeLa cells for control (ctrl), 15× phenylalanine (15× phe), and 15× tryptophan (15× tryp). $2145/2845\;{\mathrm{cm}}^{-1}$ and $2175/2940\;{\mathrm{cm}}^{-1}$ ratios are calculated to understand metabolic activities of HeLa cells between the ctrl, 15× phe, and 15× tryp groups. Multiple cell units are selected for calculating absolute intensities of $2145/2845\;{\mathrm{cm}}^{-1}$ and $2175/2940\;{\mathrm{cm}}^{-1}$ ratios between experimental groups. Reprinted with permission from Ref. 68.

Breast cancer is the most common cancer among women globally, with triple-negative breast cancer (TNBC)—an aggressive subtype representing 15% of cases—posing unique therapeutic challenges due to its metabolic dependencies on methionine and insulin.^66^ Specifically, mTOR pathway activation in TNBC is highly sensitive to methionine and insulin fluctuations, driving aberrant lipid metabolism.^67^ To dissect these metabolism dynamics, Fung et al.^67^ used conjugated $D_{2}O\text{-}\mathrm{SRS}$ and TPEF microscopy, by analyzing adipose neogenesis, lipid droplet morphology, and peroxidation under varying methionine and insulin levels in TNBC cells, as illustrated in Figs. 3(a) and 3(b). They uncovered a critical insulin-methionine interplay, suggesting therapeutic modulation of lipid metabolism could impede TNBC progression. Notably, lipid droplets emerged as phenotypic markers for subclassifying TNBC, offering a pathway toward personalized therapies.^67^

Moreover, detecting TNBC early is crucial for improving disease prognosis and optimizing treatment. Unfortunately, conventional imaging techniques fall short in providing a comprehensive differentiation of TNBC subtypes due to their limited sensitivity and inability to capture subcellular details. Li et al.^69^ developed a multi-modal imaging platform by combining $D_{2}O\text{-}\mathrm{SRS}$, TPEF, and second-harmonic generation (SHG) imaging techniques. This advanced platform allows for direct visualization and quantification of metabolic activity in TNBC subtypes at the subcellular level. Particularly, using $D_{2}O\text{-}\mathrm{SRS}$ imaging, they identified differences in *de novo* lipogenesis, protein synthesis, cytochrome C metabolic heterogeneity, and lipid unsaturation rates across TNBC subtypes. Interestingly, one specific TNBC subtype exhibited a markedly higher lipid turnover rate than others. This integrated approach establishes a sensitive platform for characterizing TNBC subtypes and tumor metabolism-related applications.^69^

Together, these studies demonstrate that $D_{2}O\text{-}\mathrm{SRS}$–based multi-modal imaging not only enables rapid, high-resolution characterization of metabolic phenotypes in TNBC but also holds potential for refining subtype classification, informing therapeutic strategies, and ultimately improving early diagnosis and prognostic assessment.

### 4.2 Cervical Cancer

The role of amino acids in cancer cell metabolism has attracted extensive attention, especially regarding how essential and non-essential amino acids jointly promote the proliferation, migration, and energy supply of cancer cells.^27^^,^^70^ Specifically, aromatic amino acids (AAAs) and branched-chain amino acids (BCAAs) not only provide energy support for protein synthesis but also drive tumor growth by regulating pathways that maintain energy supply and alleviate oxidative stress.^71^ In cervical cancer, changes in amino acid metabolism contribute to survival under stress and accelerate progression by regulating reactive oxygen species (ROS) homeostasis, lipid/protein synthesis, and immune evasion. To dissect these mechanisms, Bagheri et al. used $D_{2}O\text{-}\mathrm{SRS}$ and TPEF imaging to monitor HeLa cells exposed to excessive AAAs. Through $D_{2}O\text{-}\mathrm{SRS}$, they observed that phenylalanine/tryptophan treatment increased the production of new lipids, while reducing protein synthesis, as illustrated in Figs. 3(c) and 3(d).^68^ This finding highlights the impact of AAAs’ metabolism on cervical cancer and proposes lipid droplet phenotyping analysis as a diagnostic tool.

### 4.3 Brain Cancer

Brain cancer is a very malignant type of tumor. This malignancy is challenging to treat and frequently results in severe health complications or mortality.^72^ Alterations in metabolic activity at various tumor stages offer valuable diagnostic insights. Glioblastoma (GBM) is among the most invasive tumors, exhibiting a remarkable capacity to infiltrate healthy brain tissue. Leveraging differences in lipid and protein composition between tumor and normal cells, label-free vibrational imaging enables delineation of tumor margins.^73^ By leveraging the intrinsically elevated metabolic activity of tumor cells, $D_{2}O\text{-}\mathrm{SRS}$ detects newly synthesized lipids (${\mathrm{CD}}_{L}$) and proteins (${\mathrm{CD}}_{P}$) as metabolic contrast indicators. This principle provides a reliable means of delineating brain tumor boundaries and assessing intratumoral heterogeneity. Shi et al. established a GBM mouse model, and 25% $D_{2}O$ was administered for 15 consecutive days. Utilizing the unique metabolic spectral signatures of $D_{2}O$, they observed consistently stronger ${\mathrm{CD}}_{L}$ and ${\mathrm{CD}}_{P}$ signals in tumor tissues compared with adjacent brain regions. This metabolic contrast not only facilitated precise visualization of tumor margins but also uncovered intratumoral heterogeneity, underscoring the role of enhanced lipid and protein synthesis in glioblastoma progression.^19^ Overall, this approach revealed both tissue- and cell-type-specific metabolic patterns, offering critical insights into tumor metabolic heterogeneity. In addition, this methodology provides a foundational tool for metabolic tissue mapping in complex mammalian systems, thereby enhancing our understanding of brain cancer metabolism and informing future therapeutic strategies.

### 4.4 Colorectal Cancer

**Fig. 4:**
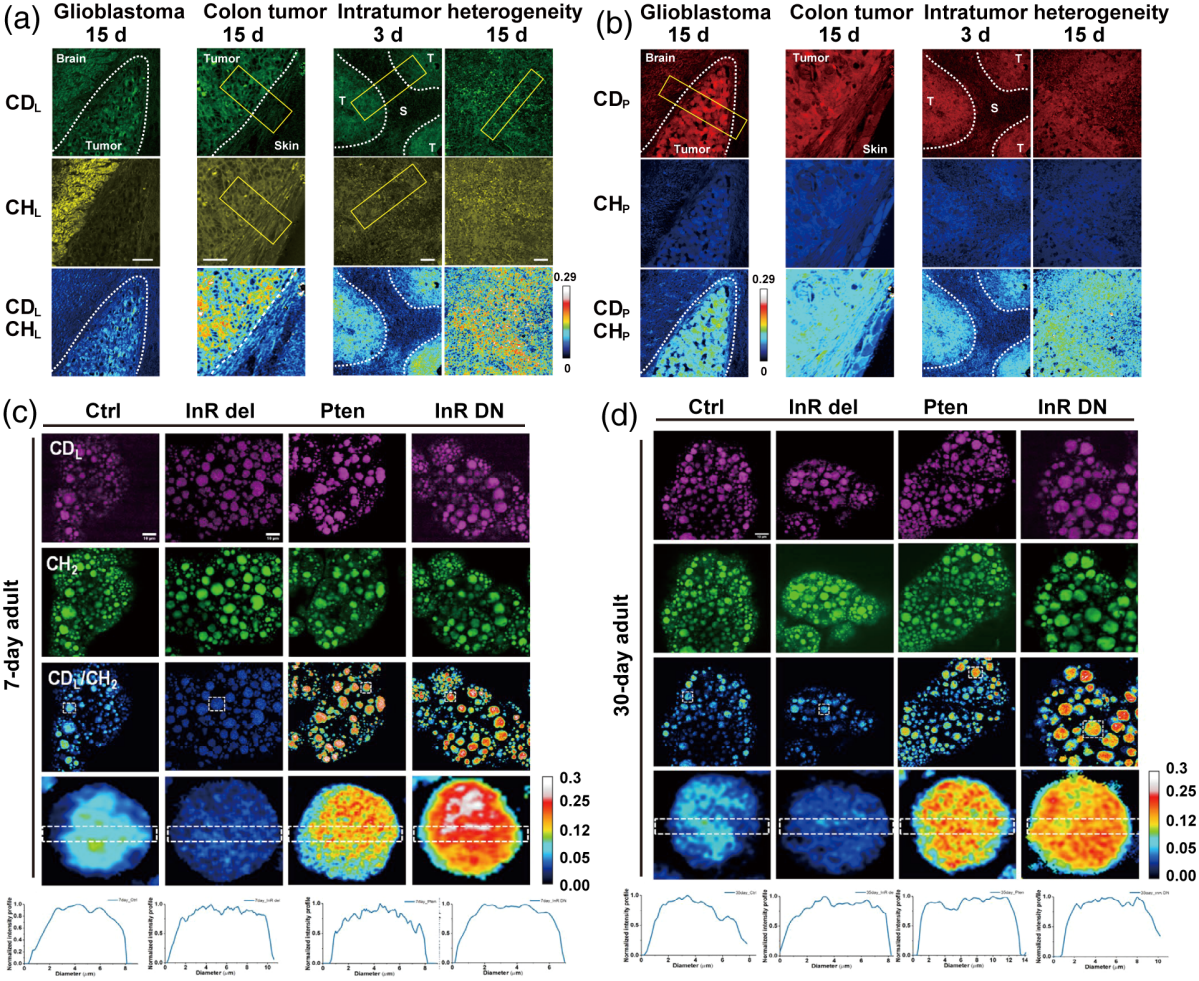
$D_{2}O\text{-}\mathrm{SRS}$ imaging of biomolecular metabolic dynamics in tumor and aging. (a), (b) $D_{2}O\text{-}\mathrm{SRS}$ microscopy identifies tumor boundaries and metabolic heterogeneity; dashed curves highlight the tumor–normal tissue boundary visualized by ${\mathrm{CD}}_{P}$ and ${\mathrm{CD}}_{L}$ signal. Intensity profile quantifies the ${\mathrm{CD}}_{P}$ signal within the yellow rectangle with x-axis showing the position along the length of the box and y-axis showing the average intensity across the width of the box; Scale bar: 20  μm. Reprinted with permission from Ref. 19. (c), (d) $D_{2}O\text{-}\mathrm{SRS}$ imaging of lipid metabolism in the fat body of mutant flies at (c) 7-day and (d) 30-day posteclosion. The ratiometric images (${\mathrm{CD}}_{L}/{\mathrm{CH}}_{2}$) display the ratio of C–D signal at $2143\;{\mathrm{cm}}^{-1}$ (newly synthesized lipids) to the signal at $2850\;{\mathrm{cm}}^{-1}$ (${\mathrm{CH}}_{2}$ stretching of lipids). The genetic downregulation of the InR/PI3K pathway (Pten, ppl-Gal4 > UAS-Pten; InR DN, ppl-Gal4 > UAS-InR DN) showed higher lipid metabolism than control, and upregulation of the InR/PI3K pathway (InR del, ppl-Gal4 > UAS-InR del) led to reduced lipid metabolism in both young and old flies. Scale bar: 10  μm. Reprinted with permission from Ref. 75.

Colorectal cancer (CRC) is the second leading cause of cancer-related mortality worldwide. Effective screening and early detection can significantly reduce the incidence of CRC, whereas timely removal of tumor lesions contributes to a decreased mortality rate.^74^ Shi et al.^19^ found that subcutaneous xenograft models of colon cancer display a protein and lipid composition similar to that of surrounding skin tissue, making them indistinguishable using label-free SRS microscopy. By contrast, $D_{2}O\text{-}\mathrm{SRS}$ microscopy delineates tumor boundaries based on the inherently higher metabolic activity of tumor tissues compared with adjacent normal tissue, by tracing deuterium incorporation from $D_{2}O$ into newly synthesized lipids and proteins, as illustrated in Figs. 4(a) and 4(b).^19^ Shi et al. also suggest that $D_{2}O$ is a more suitable probe than deuterium-labeled carbon substrates for visualizing adipogenic activity.^19^ Unlike deuterated fatty acids and glucose, $D_{2}O$ does not cause hyperglycemia, which can result from the use of deuterated glucose. In addition, $D_{2}O$ is more efficient in labeling newly synthesized lipids and is cost-effective, offering greater flexibility in detecting various metabolic activities simultaneously. This study by Shi et al. highlights the potential of $D_{2}O\text{-}\mathrm{SRS}$ microscopy as a powerful tool for metabolic imaging in oncology. The clinical utility of the tool warrants further investigation through validation in patient-derived samples and large-scale, physiologically relevant models.

Overall, $D_{2}O\text{-}\mathrm{SRS}$ has established a generalizable platform for characterizing tumor metabolic phenotypes. Its high sensitivity to alterations in lipid and protein biosynthesis, which is achieved by tracing deuterium incorporation from $D_{2}O$ into newly synthesized lipids and proteins, enables precise, real-time *in vivo* detection and delineation of tumor margins. By integrating spatial, temporal, and biochemical information, $D_{2}O\text{-}\mathrm{SRS}$ represents a powerful tool in cancer research, particularly in studies aiming to correlate metabolic phenotypes with therapeutic vulnerabilities. Future investigations that integrate $D_{2}O\text{-}\mathrm{SRS}$ with omics-based approaches and functional imaging modalities may further elucidate the molecular mechanisms driving tumor-specific metabolic dependencies, thereby contributing to the advancement of precision oncology.

### 4.5 Drug Responses in Cancer

Due to the heterogeneity of stress responses among individual cells or organelles, analyses at single-cell or subcellular resolution are critical for understanding how cells respond to drugs and therapies.^76^ For example, tetrazolium-salt–based assays, such as the 3-(4,5-dimethylthiazol-2-yl)-2,5-diphenyltetrazolium bromide (MTT) assay, are commonly used to assess compound toxicity due to their simplicity, sensitivity, and low cost. However, these methods have several limitations. They cannot distinguish between cytotoxic (cytocidal) and cytostatic (antiproliferative) effects, and they are ineffective in evaluating drugs that target organelle metabolism, such as mitochondrial function.^77^ In addition, these assays are less reliable when assessing samples with low cell numbers or when metabolic changes, oxidative stress, or alterations in oxidoreductase activity compromise the results.^78^^,^^79^

Confocal fluorescence microscopy is another widely used technique for monitoring real-time changes in drug responses at the single-cell level. However, it typically requires exogenous labeling with fluorophores or dyes, and the data obtained are often limited by the fact that only a few molecules can be labeled simultaneously using fluorescent probes.^80^ To overcome these limitations, Hekmatara et al.^77^ employed $D_{2}O\text{-}\mathrm{SRS}$ to investigate drug responses at the single-cell and subcellular level. By monitoring shifts in C–D bond vibrations as indicators of macromolecular synthesis, they were able to dynamically track protein and lipid metabolic activities in MCF-7 cancer cells and yeast exposed to rapamycin. This approach provides a promising strategy for elucidating organelle-specific metabolic dynamics and revealing drug-tolerant subpopulations that are often overlooked by conventional population-based assays.^77^

## 5 Detection of Biomolecular Metabolic Dynamics in Aging and Related Diseases

Aging is characterized by the gradual decline of cellular function, driven by imbalances in protein homeostasis and lipid metabolism. These processes are tightly regulated in youth but deteriorate with age, contributing to diseases like Alzheimer’s and cardiovascular disorders.^81^ Traditional methods struggle to capture dynamic metabolic fluxes, but $D_{2}O\text{-}\mathrm{SRS}$ enables real-time tracking of synthesis and degradation.^82^ For example, Li et al.^83^ used $D_{2}O\text{-}\mathrm{SRS}$ in aging *Drosophila* to show that protein turnover declines earlier than lipid metabolism; they found that lipid metabolic activity significantly declined in 35-day-old *Drosophila*. Notably, lipid droplets—organelles storing fatty acids—exhibited size-dependent metabolic activity, suggesting distinct roles in aging.^83^ Li et al.^84^ also observed metabolic changes in the ovaries of aging *Drosophila*. $D_{2}O\text{-}\mathrm{SRS}$ imaging revealed that protein synthesis was more active during the early developmental stages, whereas lipid turnover significantly increased in later stages. As the flies entered old age (35 days), both protein and lipid turnover activities declined, especially in the germline stem cell microenvironment. Notably, an accumulation of unsaturated lipids in trophoblasts and oocytes in aged *Drosophila* suggests that these lipids play a key role in oocyte maturation. In addition, changes in mitochondrial morphology and a significant increase in cytochrome c accumulation were observed, highlighting the profound impact of aging on cellular metabolism and organ function.^84^ These findings in the *Drosophila* ovary underscore the tissue-specific nature of metabolic decline. Beyond reproductive tissues, systemic lipid metabolism is tightly regulated by diet and signaling pathways, offering additional insights into aging mechanisms.

Given the age-related decline in lipid turnover observed in *Drosophila* ovaries, Li et al.^75^ investigated whether dietary interventions could modulate systemic lipid metabolism. Using $D_{2}O\text{-}\mathrm{SRS}$, they quantified lipid turnover in the fat body of flies under varying diets and insulin signaling conditions. They found that calorie restriction, low-protein diets, and moderately high-protein, high-sucrose diets all increased lipid turnover across various ages in fruit flies; see Figs. 4(c) and 4(d). By contrast, enhanced insulin signaling reduced lipid turnover and shortened lifespan. This study provides a method for observing spatiotemporal changes in lipid turnover and enhances our understanding of how lipid metabolism, diet, and aging are interconnected.^75^

In aging and neurodegenerative diseases such as Alzheimer’s, the accumulation of lipid droplets (LDs) in the brain is a significant pathological feature, yet the underlying cellular and molecular mechanisms remain unclear. By utilizing $D_{2}O\text{-}\mathrm{SRS}$ to trace newly synthesized lipids, Li et al.^85^ found that heightened lipogenesis and impaired lipid turnover occurred within LDs in tauopathy fly brains and human neurons derived from induced pluripotent stem cells (iPSCs), and that the transfer of unsaturated lipids from tauopathy iPSC neurons to microglia induces LD accumulation, oxidative stress, inflammation, and impaired phagocytosis. This work provides direct evidence of native, aberrant LD accumulation in tauopathy brains.^85^ This direct visualization of neuron-microglia lipid exchange provides critical insights into how lipid dysregulation exacerbates neurodegeneration. Moreover, the study’s use of human induced pluripotent stem cell (iPSC)-derived neurons underscores $D_{2}O\text{-}\mathrm{SRS}$’s applicability to human models, bridging preclinical and clinical research. These findings exemplify the power of $D_{2}O\text{-}\mathrm{SRS}$ to resolve spatially and temporally dynamic metabolic processes, positioning it as a pivotal tool for both mechanistic discovery and therapeutic development in neurodegenerative diseases.

In conclusion, $D_{2}O$-based detection offers a non-carbon isotope tracing method that minimally perturbs endogenous metabolic processes, thereby rendering it highly suitable for studying *de novo* biosynthesis. These advantages establish the $D_{2}O\text{-}\mathrm{SRS}$ method as a powerful and adaptable tool for investigating metabolic activities across various domains, including aging, cancer, and infectious diseases. Notably, the $D_{2}O\text{-}\mathrm{SRS}$ microscope exhibits exceptional sensitivity, even at low $D_{2}O$ concentrations, and demonstrates minimal toxicity, underscoring its potential for clinical metabolic monitoring in future translational applications.

## 6 Discussion

### 6.1 Broad Applications of D_2_O Probe

Metabolism plays a crucial role in the occurrence, development, and outcome of diseases, thus becoming one of the key focus areas in precision medicine.^86^^,^^87^ Accurate capture of metabolic characteristics is of vital importance for the development of treatment plans. The $D_{2}O\text{-}\mathrm{SRS}$ microscopy technique combines stable isotope detection with Raman scattering technology, achieving high-sensitivity and high-resolution metabolic imaging at the subcellular level. This makes it a powerful and versatile tool for characterizing the metabolic activity of living organisms. Meanwhile, $D_{2}O\text{-}\mathrm{SRS}$ can monitor metabolic changes in real time, providing immediate feedback on dynamic physiological and pathological states.^65^

Beyond metabolism, water permeation kinetics, regulated by the integrity of the skin barrier, can be assessed using $D_{2}O$ as a skin probe. In skin research, $D_{2}O$ is also used as a permeation probe to assess the integrity of the skin barrier. This method, through pixel-level spectral information extraction, can distinguish endogenous and exogenous hydrogen bonds, thereby monitoring the distribution and dynamics of water in skin tissue. This is particularly important for patients with diseases such as eczema and psoriasis, as it can assist doctors in selecting appropriate drugs or moisturizers and providing real-time feedback on therapeutic efficacy. Taken together, $D_{2}O\text{-}\mathrm{SRS}$ offers a potential means for the early identification of barrier alterations and metabolic changes, underscoring its promise in both research and clinical contexts.^88^^,^^89^

### 6.2 Current Challenges and Limitations of D_2_O-SRS

$D_{2}O$ has quantifiable characteristics in aspects such as tissue perfusion, hemodynamics, and cellular metabolism, demonstrating its wide applicability in clinical practice. $D_{2}O$ can also be used as an intravascular neurointerventional contrast agent. However, despite the numerous advantages of $D_{2}O\text{-}\mathrm{SRS}$, its widespread application still faces several limitations.

First, $D_{2}O$ itself has certain toxicity, and the toxicity thresholds of $D_{2}O$ in different types of tissues, cells, and even different species have not been systematically clarified. Further experiments are needed to confirm these thresholds. Currently, there is a lack of systematic studies to define the relationship between $D_{2}O$ concentrations in the body, exposure time, and physiological metabolism. Care must be taken to handle the interference of $D_{2}O$ on intrinsic metabolic activity. The concentration of $D_{2}O$ used in most experiments (e.g., 1% to 5%) is generally considered a low-toxicity level, but the safe dose for specific tissues or disease states still needs individual assessment. Second, $D_{2}O$ has limited specificity and cannot target specific metabolic pathways or molecular types. In complex metabolic contexts, to improve the accuracy of signal recognition, it is necessary to combine other probe labeling methods or integrate multi-omics technologies (transcriptomics, proteomics, metabolomics) for multi-dimensional joint analysis, thereby improving the accuracy and systematicity of data interpretation. Third, the current $D_{2}O\text{-}\mathrm{SRS}$ technology lacks standardized data processing procedures and automated analysis tools, especially in extracting quantitative parameters such as steady-state and conversion rates from dynamic maps. This limits its reproducibility and throughput efficiency in large-scale histological research and clinical routine applications. Fourth, although $D_{2}O\text{-}\mathrm{SRS}$ shows promising prospects in microbial research, its throughput is still relatively low, making it difficult to efficiently handle complex environmental samples. $D_{2}O\text{-}\mathrm{SRS}$ still lacks research in areas such as degenerative diseases (such as neurodegenerative diseases, muscle atrophy), drug sensitivity screening, and disease heterogeneity assessment. These fields can be advanced by expanding probe design, improving image processing algorithms, and integrating artificial intelligence-assisted analysis.

### 6.3 Technological Innovations Addressing Current Issues

Recent advances offer feasible strategies to address its current limitations. These innovations can be broadly categorized into improvements in sensitivity, resolution, and deep learning algorithms.^90^ To push the detection limit to sub-mM levels, researchers have developed advanced strategies, such as microsecond-scale vibrational imaging and electron pre-resonance SRS.^91^^,^^92^ Liao et al. achieved rapid Raman spectral acquisition at 32  μs using a tuned amplifier array and with close to shot-noise limited detection sensitivity. Furthermore, Wei and Min introduced electron pre-resonance SRS to achieve sub-μM sensitivity on chromophores.^93^ To effectively reveal the complex metabolic structures and functional changes within cells, Lin et al. proposed a new ultrasensitive reweighted visible stimulated Raman scattering (URV-SRS) technique. This method integrates innovative optical design with computational denoising algorithms, which together significantly enhance both the sensitivity and resolution of imaging. As a result, it enables researchers to clearly observe subtle changes in cellular metabolism at the nanoscale.^90^

In terms of technological progress in resolution, super-resolution SRS can visualize processes such as intracellular lipid synthesis, providing a new perspective for revealing the mechanisms of metabolic changes at the subcellular scale.^94^ The development of computational deconvolution algorithms surpassed the limitations imposed by the numerical aperture of the imaging objective lens and the molecular scattering cross-section.^19^ Specifically, Jang et al.^95^ developed a novel deconvolution algorithm—point deconvolution based on Adam optimization (A-PoD). Utilizing A-PoD coupled with an SRS microscope, it becomes possible to distinctly distinguish the spatial distribution of newly synthesized versus existing lipids in living cells and tissues. By mapping the structural domains of old and new lipids in individual lipid droplets, they gained valuable insights into lipid metabolism at the nanoscale.^95^ Meanwhile, by combining hyperspectral image unmixing technology with deep learning classification models, the recognition accuracy of metabolic features and the efficiency of multi-species identification can be improved, especially for the identification of unknown metabolic pathways.^96^^,^^97^

The emergence of ultra-fast SRS microscopic imaging has also promoted the three-dimensional tracking ability of organelle metabolic dynamics during the development of organisms.^98^ Moreover, the combination and miniaturization progress of SRS microscopes and endoscopes also provide possibilities for their *in vivo* applications, such as rapid identification of tumor boundaries and targeted resection during surgery.^70^

### 6.4 Future Prospects and Clinical Translation of D_2_O-SRS

$D_{2}O\text{-}\mathrm{SRS}$, as a forward-looking metabolic imaging platform, is breaking through the limitations of traditional optical imaging and chemical labeling in multiple directions. This technology not only supports molecular metabolic visualization research but also provides new tools for the assessment of individualized treatment response in precision medicine. However, the process of its transition from laboratory research to clinical practice still needs to address key challenges, including the establishment of imaging standards, the control of the toxicity of heavy water intake, and the establishment of a large-sample clinical validation mechanism. With the further advancement of technology, $D_{2}O\text{-}\mathrm{SRS}$ is expected to develop into a “metabolic microscope” that serves the dynamic analysis of life processes and the precise diagnosis of diseases, promoting the transformation and implementation of basic metabolic research into clinical applications.

## Data Availability

Data sharing is not applicable to this article, as no new data were created or analyzed.
